# Auxin response factor gene family in *Brassica rapa*: genomic organization, divergence, expression, and evolution

**DOI:** 10.1007/s00438-012-0718-4

**Published:** 2012-08-24

**Authors:** Jeong-Hwan Mun, Hee-Ju Yu, Ja Young Shin, Mijin Oh, Hyun-Ju Hwang, Hee Chung

**Affiliations:** 1 Department of Agricultural Biotechnology, National Academy of Agricultural Science, Rural Development Administration, 150 Suin-ro, Gwonseon-gu, Suwon, 441-707 Korea; 2 Department of Life Sciences, The Catholic University of Korea, Bucheon, 420-743 Korea

**Keywords:** *Brassica rapa*, Auxin response factor, Genome organization, mRNA sequencing, Evolution

## Abstract

Completion of the sequencing of the *Brassica rapa* genome enabled us to undertake a genome-wide identification and functional study of the gene families related to the morphological diversity and agronomic traits of *Brassica* crops. In this study, we identified the auxin response factor (*ARF*) gene family, which is one of the key regulators of auxin-mediated plant growth and development in the *B. rapa* genome. A total of 31 *ARF* genes were identified in the genome. Phylogenetic and evolutionary analyses suggest that *ARF* genes fell into four major classes and were amplified in the *B. rapa* genome as a result of a recent whole genome triplication after speciation from *Arabidopsis thaliana*. Despite its recent hexaploid ancestry, *B. rapa* includes a relatively small number of *ARF* genes compared with the 23 members in *A. thaliana*, presumably due to a paralog reduction related to repetitive sequence insertion into promoter and non-coding transcribed region of the genes. Comparative genomic and mRNA sequencing analyses demonstrated that 27 of the 31 *BrARF* genes were transcriptionally active, and their expression was affected by either auxin treatment or floral development stage, although 4 genes were inactive, suggesting that the generation and pseudogenization of *ARF* members are likely to be an ongoing process. This study will provide a fundamental basis for the modification and evolution of the gene family after a polyploidy event, as well as a functional study of *ARF* genes in a polyploidy crop species.

**Electronic supplementary material:**

The online version of this article (doi:10.1007/s00438-012-0718-4) contains supplementary material, which is available to authorized users.

## Introduction

Plant growth and development is regulated both by internal and external factors, including plant hormones and environmental stimuli. Auxin is one of the plant hormones that affect the genes involved in development processes at the cellular, tissue, and organ levels. Auxin treatment is known to increase the transcription of three early auxin response gene families, including SMALL AUXIN UP RNA (*SAUR*), Gretchen Hagen 3 (*GH3*), and indole-3-acetic acid-inducible gene (*Aux/IAA*), by modulating the interaction of transcription factors with auxin response elements (AuxREs) of the affected genes (reviewed in Hayashi 2012). A number of putative AuxREs have been defined within the upstream promoter regions of primary/early auxin responsive genes, such as one or more copies of a conserved motif, TGTCTC, or some variation of the motif (i.e., TGTCCC or TGTCAC), which confer auxin responsiveness (Ulmasov et al. 1997; Ulmasov et al. 1995). The transcription factors that bind specifically to these motifs are called auxin response factors (ARFs).

ARF is one of the plant protein families well known for their role in auxin-mediated responses. Most ARFs consist of three unique domains. The N-terminal region is a B3 DNA-binding domain and the C-terminal region has protein–protein interaction domains which are similar to those found in the C-terminal of Aux/IAAs and allow the dimerization of ARFs or ARF and Aux/IAA proteins. The variable middle region contains an activation or repression domain (reviewed in Guilfoyle and Hagen 2007). It has been reported that the ARF proteins are encoded by a larger gene family in *Arabidopsis thaliana* (Hagen and Guilfoyle 2002), maize (Xing et al. 2011), rice (Wang et al. 2007), poplar (Kalluri et al. 2007), and tomato (Kumar et al. 2011). A genome-wide comparison of the *ARF* gene family suggested that the whole genome and chromosomal segment duplication events are primarily responsible for the expansion of *ARF* genes in the plant genomes. In addition, the tandem duplication is an additional contributor that occurred in *A. thaliana* (Finet et al. 2010; Wang et al. 2007; Xing et al. 2011). Expression analysis demonstrated that these genes are, in general, transcribed in a wide variety of tissues and organs, with the exception of the *ARF* gene cluster on *A.* *thaliana* chromosome 1, which appears to be restricted to embryogenesis and seed development (Okushima et al. 2005).

Genetic studies have identified the well-established role of *ARF* genes in plant growth and development in *A. thaliana*. Mutations to *ARF* genes resulted in changes in the vascular strands and embryo axis formation (*AtARF5*) (Hardtke and Berleth 1998), suppression of hookless phenotype and hypocotyls bending (*AtARF1* and *AtARF2*) (Ellis et al. 2005; Li et al. 2004), increased size and weight of seeds (*AtARF2*) (Schruff et al. 2005), abnormal gynoecium patterning (*AtARF3*) (Nishimura et al. 2005), abnormal development of floral organs and leaves (*AtARF3* and *AtARF4*) (Finet et al. 2010), impaired hypocotyl response to blue light, growth, and auxin sensitivity (*AtARF7*) (Fukai et al. 2006; Sorin et al. 2005), and changed auxin homeostasis (*AtARF8*) (Goetz et al. 2006). Moreover, a double mutation showed a more severe phenotype than a single mutation, suggesting the unique and overlapping function of the *ARF* gene family members (*AtARF7* and *AtARF19*) (Okushima et al. 2005). In addition, there is a growing body of information on the posttranscriptional regulation of *ARF* transcript abundance by micro-RNAs (miRNA) and *trans*-*acting*-small interfering RNAs (ta-siRNA). While *AtARF6* and *8* are targets of miR167, and *AtARF10*, *16*, and *17* are targeted by miR160, *AtARF2*, *3*, and *4* are targets of TAS3 ta-siRNAs in *A. thaliana* (Guilfoyle and Hagen 2007). Mutation of the epigenetic regulators caused alterations in seed germination or leaf development, juvenile to adult phase changes, or heteroblasty (Fahlgren et al. 2006; Liu et al. 2007; Vaucheret et al. 2004).

*Brassica rapa* is of importance for agriculture and human nutrition. It is a member of the Brassicaceae family, like *A. thaliana*, and includes vegetables as well as oil crops. There are three well-defined groups of the cultivated species based on their morphological characteristics. The leafy type includes “Kimchi” cabbage (Korean heading form), Chinese cabbage, Pak choi, and celery mustard. The turnip type is comprised of turnip, rapini, and turnip broccoli. These two types are important as vegetable sources worldwide and are also useful as fodder. The oil type species are yellow sarson and toria, which are important oil crops in India and Canada. The diverse morphological variants of the species appear to be linked to the genomic changes associated with polyploidy (Lukens et al. 2004). Selection for different characteristics or hybridization between intraspecific types after domestication since 2,000 BC (Plant Biosafety Office 1999) might serve as an additional source of the morphological diversity of agronomically important cultivars. Morphological changes to the plant during genome evolution or domestication provides clues as to the evolutionary genetics of the genes related to structural diversification and the origin of crop species. One approach to understanding the fundamental basis of morphological diversity is the genomic evaluation of genes that are potentially involved in plant morphology, such as auxin response factors. Recent completion of sequencing of the euchromatic gene space of *B. rapa* allows for the genome-level identification of specific gene families in the genome.

A draft genome sequence of *B. rapa*, covering approximately 284 Mb of the genome, was assembled by Illumina GA short-read sequences and Sanger BAC-end sequences (The *Brassica* *rapa* Genome Sequencing Project Consortium 2011). This assembly was estimated to cover more than 98 % of the gene space. After the annotation and analysis, 41,174 protein-coding genes were identified in the genome, which are roughly 1.5 times as many genes as are found in *A. thaliana* (27,411, TAIR10), a difference that can be attributed to the whole-genome triplication (WGT, mesopolyploidy) thought to have occurred approximately 13 million years ago (Mya), followed by substantial gene loss (fractionation). This is consistent with our previous reports regarding BAC and A3 chromosome sequencing (Mun et al. 2009a, 2010). In particular, several hormone-related gene families, including auxin-related genes, were expanded or over-retained in the *B. rapa* genome compared with other gene families, which may contribute to the development of morphological variants of the species. The mesohexaploidy of *B. rapa* offers an opportunity to study the retention, subfunction, and neofunction of genes in the triplicated genome, as well as variation of the structure and function of the gene families. However, analyses of divergence as well as variation in the retained gene family have not yet been studied.

The main objective of this study is to identify *ARF* gene candidates of *B. rapa* (*BrARFs*) in the draft genome sequences. To the best of our knowledge, we determined the sequence characteristics of the *ARF* genes and examined their genomic distribution, along with phylogenetic and evolutionary analysis of the gene family to gain insight into the origin, divergence, and evolution of *ARF* genes in *B. rapa*. In addition, differentiation of expression between the paralogs in response to auxin treatment in seedlings, as well as during anther and pistil development, was investigated using next-generation mRNA sequencing technology (RNA-Seq). Understanding of the genomic organization of *ARF* genes in *B. rapa* will allow genome-level insights into the divergence, variation, and evolution of the auxin-related gene family, and will inform genetic and breeding strategies to engineer morphological plasticity in *Brassica* crops. In addition, this study will be beneficial to the understanding of the influence of polyploidy in the evolution of a gene family.

## Materials and methods

### Identification of *ARF* gene family in the *B. rapa* genome

We used 41,174 gene models of *B. rapa* ssp. *pekinensis* cv. *Chiifu* to search for *ARF* gene candidates in the genome. All publicly known *A. thaliana ARF* genes (*AtARF1* to *AtARF23*) were used in the initial protein queries and candidate genes were identified based on a BLASTP search at a cut-off value of <*E* ^−20^ (Altschul et al. 1997). To ensure that there were no additional related genes missing from the gene models, a TBLASTN search was also used against the 284 Mb assembled genome sequences at a cut-off value of <*E* ^−10^. A conserved domain and motif search was performed using the Conserved Domain Database (CDD) of the National Center for Biotechnology Information (NCBI) to confirm each candidate protein sequence as an auxin response factor protein (Finn et al. 2008). To identify expressed sequence tag (EST) matches of the candidate *ARF* genes of *B. rapa*, 147,217 EST sequences of *B. rapa* ssp. *pekinensis* cv. *Chiifu* that we generated were used (Yu et al. 2011). All ESTs were compared with the coding sequences of candidate *ARF* genes. We considered ESTs to have a genome match if more than 90 % of the sequences matched with at least 95 % identity in a BLASTN search. If one EST matched with multiple candidate genes, only the gene with the best alignment score was considered.

A promoter search was performed to identify auxin response elements within the approximately 1 kb region upstream from the 5′-untranslated region (5′-UTR) of the *BrARF* genes, based on search of the PLACE database (www.dna.affrc.go.jp/PLACE). To determine whether specific repetitive elements drive the sequence divergence of *BrARF*, the tandem repeat (TR), inverted repeat (IR), transposable element (TE), low complexity repeat (LCR), and simple sequence repeats (SSRs) were also investigated in the region 1 kb upstream of the 5′-UTR to the 3′-UTR of the genes. Since the draft *B. rapa* genome did not provide information on the 5′- and 3′-UTR regions of the gene models, we used in-house trained FGENESH (www.softberry.com) to predict the 5′- and 3′-UTR regions of the *BrARF* genes. For those genes that have an intergenic sequence shorter than 1 kb from the end of the former gene, only the region between the 3′-UTR of the former gene to the 5′-UTR of the *BrARF* genes was considered as a putative promoter region. TRs and IRs were searched using Tandem Repeat Finder version 4.04 (Benson 1999) and Inverted Repeats Finder version 3.05 (Warburton et al. 2004), using default parameter values, respectively. TEs and LCRs were detected by RepeatMasker (http://www.repeatmasker.org) using *B. rapa* repetitive sequences. For identification of SSRs longer than 20 bp with mono- to deca-nucleotide motifs, the SSRIT (Temnykh et al. 2001) was used.

### Phylogenetic analysis of the sequences

For phylogenetic reconstruction of the *ARF* gene family, we downloaded the sequenced plant genomes, as of November 2011, from the NCBI Entrez Genome database (http://www.ncbi.nlm.nih.gov/genome/PLANTS/PlantList.html). We identified putative *ARF* genes from the sequenced genomes, which included *A. thaliana* (23), papaya (13), cucumber (16), strawberry (17), soybean (54), poplar (36), castor bean (18), cacao (19), grape (19), *Brachypodium distachyon* (24), rice (28), sorghum (25), and maize (37), based on a BLASTP search as described earlier. Amino acid sequences of the *ARF* genes were aligned using ClustalW (Thompson et al. 1994), using the default options, and the alignment was manually corrected using the alignment editor Jalview (Waterhouse et al. 2009). Aligned sequences were trimmed at both ends to eliminate regions of poor alignment. Phylogenetic trees were constructed using the neighbor-joining (NJ) and the maximum likelihood (ML) methods in MEGA5 (Tamura et al. 2011). The stability of tree nodes was tested by bootstrap analysis with 1,000 replicates.

### Genome block comparison and synonymous substitution rate analysis of homologous sequences between *B. rapa* and *A. thaliana*

Syntenic regions between the *B. rapa* and *A. thaliana* genomes were identified by a proteome comparison based on BLASTP analysis (Altschul et al. 1997). The entire proteomes of the two genomes were compared, and only the top reciprocal BLASTP matches per chromosome pair were selected (minimum of 50 % alignment coverage at a cutoff of <*E* ^−20^). Chromosome scale synteny blocks were inferred by visual inspection of dot-plots using DiagHunter with parameters as described in previous reports (Cannon et al. 2003; Mun et al. 2009a). Assignment of 24 building blocks of ancestral karyotype (*AK*) to both genomes was carried out according to previous comparative genome mapping studies (Lysak et al. 2006; Schranz et al. 2006), and homologous gene pairs between the genomes were illustrated by Circos (Krzywinski et al. 2009).

The timings of a duplication event or divergence of homologous genes and selective pressure on duplicated genes were estimated by calculating the synonymous (*K*_s_) and non-synonymous substitution (*K*_a_) per site between homologous genes. To calculate the *K*_s_ and *K*_a_ values, the protein sequences of genes were aligned and the resulting alignment was used as a reference to align the nucleotide sequences. After the removal of gaps, the *K*_s_ and *K*_a_ values were determined using the maximum likelihood method implemented in the CODEML (Goldman and Yang 1994) module of the PAML (Yang 2007) package, as previously described (Mun et al. 2009b). The *K*_s_ age distribution was represented at an interval of 0.1 and the calculation of divergence time was based on the neutral substitution rate of 1.5 × 10^−8^ substitutions per site per year for chalcone synthase (*Chs*) (Koch et al. 2000).

### Plant material and library construction for mRNA sequencing

Seeds of *B. rapa* ssp. *pekinensis* cv. *Chiifu* were surface-sterilized in 12 % sodium hypochlorite and were germinated on 0.5× Murashige and Skoog (MS) agar plates (0.7 %) in a growth chamber at 22 °C with a 16 h light/8 h dark cycle and 60 % humidity. Seedlings, 5 days after germination, were placed on 0.7 % agar plates containing 1 μM 1-naphthalene acetic acid (NAA) in 0.5× MS agar media for 0, 0.5, 1, and 2 h under the same growth conditions as described earlier and then were harvested. To collect floral tissues, 1-month-old plants were vernalized for 4 weeks at 4 °C and then were grown in a growth chamber. After inflorescences induction, the anthers and pistils at floral development stages 9–13 were collected. Floral development stage was determined according to that of *A. thaliana* (Smyth et al. 1990; Yu et al. 2005).

Total RNA was isolated from the plant tissues using the GeneAll HybridR^+^ kit (GeneAll, Seoul, Korea), and messenger RNA was purified using the TruSeq^TM^ RNA sample preparation kit (Illumina, San Diego, CA) following the manufacturer’s instruction. Purified mRNA was chemically fragmented to 200 bp fragments and the first- and second-strand cDNAs were synthesized, followed by end repair and index adapter ligation. Next, the resulting libraries were pooled with up to 12 samples per flow cell lane for cluster generation on the Illumina flow cell using the cBot.

### mRNA sequencing and data analysis

Gene expression analysis (three samples per treatment or tissue as biological replicates) was conducted by Illumina RNA-Seq technology. The library clusters were sequenced on the Illumina HiSeq1000 sequencer using the TruSeq™ SBS kit v3-HS to generate paired-end sequences (2×100 bp). Real-time analysis and base calling were performed using the CASAVA 1.8.2 software package (Illumina). The approximate number of reads collected from seedling, anther, and pistil libraries were 38, 25, and 24 million reads, respectively, with a minimum Illumina quality score of 31.

The paired-end sequence reads were aligned to the *B. rapa* ssp. *pekinensis* cv. *Chiifu* reference gene models using CLC Assembly Cell 3.2 (CLC Bio, Aarhus, Denmark), with a minimum similarity fraction of 0.9, a minimum length fraction of 0.9, a maximum of two mismatches, and paired-read mode options. The reads that mapped to multiple locations were excluded. The resulting mapped reads were normalized using the TMM (Trimmed Mean of M component) method for each gene to report the expression of genes. The TMM process estimates scale factors from the raw data that can be used in downstream statistical analysis procedures such as the detection of differential expression (Robinson and Oshlack 2010). The data of three biological replicates were pooled and the average expression levels of *BrARF* genes were extracted and analyzed.

## Results

### Identification of *BrARF* genes in the *B. rapa* genome

**Fig. 1:**
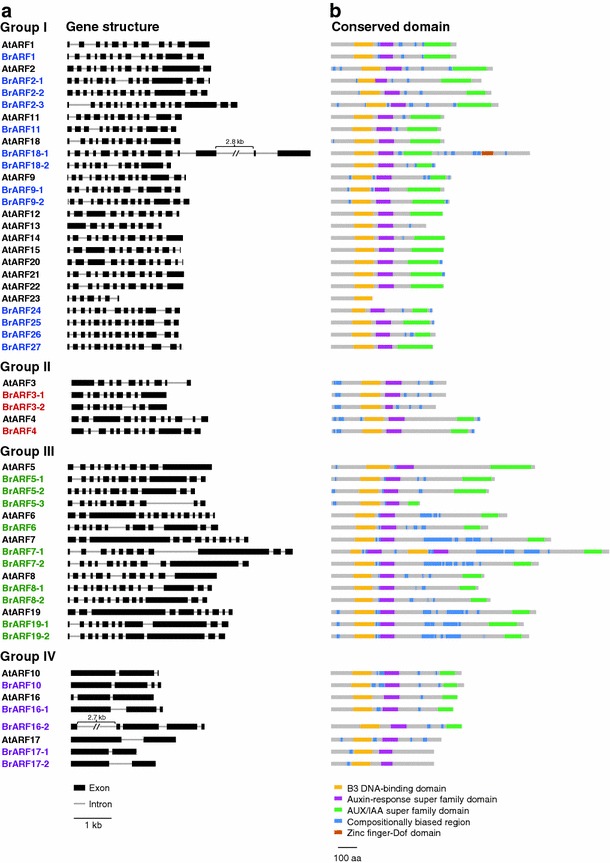
Gene structure and conserved protein domain of *BrARF* genes. Exon–intron organization of the *BrARF* genes and conserved domains identified in the encoded protein were displayed together with those of their *AtARF* homologs. **a** Exon–intron structure organization of *BrARF* and *AtARF* genes. The exons and introns are represented by *black boxes* and *gray lines*, respectively. **b** Conserved domain structure of BrARF and AtARF proteins. The conserved domains are denoted by *colored boxes*

The *BrARF* genes were identified based on a BLAST search of all reference gene models against the *A. thaliana* *ARF* gene family, followed by an hmmpfam search through the CDD database. A total of 31 *BrARF* genes were identified from the genome (Table 1). They were distributed on all 10 chromosomes and no tandem clusters of the genes in 1 Mb intervals were identified, unlike the cluster of *AtARF* genes on chromosome 1 of *A. thaliana* (*AtARF 12*–*15* and *20*–*23*). The gene structure of *BrARFs* is similar to that of *AtARFs* with an average of 11 exons in the *BrARF* genes. However, *BrARF10*, *16*-*1*, *16*-*2*, *17*-*1*, and *17*-*2* have only 2–5 exons, like their *A. thaliana* homologs (Fig. 1). The genes encode weak acidic proteins ranging in size from approximately 60 to 121 kDa. Like other transcription factors, BrARFs contain conserved domains that can function in DNA binding. All the BrARFs consist of an N-terminal plant-specific B3 DNA-binding domain and an auxin response super family domain at the middle region. However, a C-terminal AUX/IAA super family domain was missing in several proteins, including BrARF3-1, 3-2, 17-1, and 17-2. In *A. thaliana*, AtARF3, 13, and 17 also lack the C-terminal AUX/IAA domain, which functions as a dimerization domain with AUX/IAA proteins. In addition, *AtARF23* is a partial-length gene with a stop codon in its B3 DNA-binding domain (Guilfoyle and Hagen 2007), but no such truncated genes were identified in the *B. rapa* genome. In silico analysis of gene expression indicated that 18 genes (58 %) have EST support, with an average of 3.1 ESTs per expressed *BrARF* gene. These genes were expressed in a narrow range of cDNA libraries, including those constructed from root, floral tissues, or stress-induced condition, suggesting the specialized expression of *BrARF* gene members in *B. rapa* plants. The most abundantly expressed gene was *BrARF2*-*2*, which displayed 9 ESTs, both in vegetative and floral tissues. Alternative transcription was observed in *BrARF6*, of which two ESTs had lengths of an additional 36 bp due to differential splicing of the 11th exon–intron junction compared with the coding sequence. The remaining 13 genes had no ESTs collected. They might play a unique role in specific tissue or developmental conditions, or be silenced due to redundancy.

**Table 1:** *ARF* gene family in *B. rapa*, along with their molecular details and relevant genomic information

Gene			Deduced polypeptide			Genomic locus			
Name	NCBI accession	ORF length (bp)	Length (aa)	MW^a^ (kDa)	p*I* ^b^	Chr^c^	Position (Mbp)	ESTs accession	Expressed tissue
*BrARF1*	JN979458	1,998	665	73.8	6.35	A1	12.89	EX099525	Root, 1, 3, and 7 weeks old
								EX100479	Root, 1, 3, and 7 weeks old
*BrARF2*-*1*	JN979459	2,397	798	89.7	6.14	A2	26.09	EX045932	Floral bud, >2 mm in size
*BrARF2*-*2*	JN979460	2,556	851	94.6	6.62	A6	13.65	EX022541 EX130880 EX053593 EX053706 EX048281 EX049453 EX133991 EX030488 EX031313	Root, mixed stage and treatment Root, mixed stage and treatment Floral bud, <2 mm in size Floral bud, <2 mm in size Floral bud, <2 mm in size Floral bud, <2 mm in size Root, mixed stage and treatment Callus Callus
*BrARF2*-*3*	JN979461	2,667	888	99.0	6.27	A9	34.09	EX036393 EX019342 EX059199	Floral bud, >2 mm in size Whole plant, cold treated Whole plant, salt treated
*BrARF3*-*1*	JN979462	1,818	605	66.3	6.67	A5	18.30	EX119165 KFXT-027B01 KFXT-036B09 KFXT-068E04	Cotyledon, greening stage Whole plant, heat treated Whole plant, heat treated Whole plant, heat treated
*BrARF3*-*2*	JN979463	1,659	552	60.5	6.77	A4	16.61	EX100356 EX135437 KFXT-020A07 KFXT-068C01	Root, 1, 3, and 7 weeks old Root, mixed stage and treatment Whole plant, heat treated Whole plant, heat treated
*BrARF4*	JN979464	2,277	758	83.8	6.71	A10	8.29		
*BrARF5*-*1*	JN979465	2,604	867	96.1	6.05	A7	9.13	EX018749 EX038312 KFFO-054C04	Whole plant, cold treated Floral bud, >2 mm in size Floral organ, mixed stage
*BrARF5*-*2*	JN979466	2,511	836	92.8	5.71	A8	2.93	KFFO-011H12 KFFO-024F08 KFFO-044H05 KFFO-094D08	Floral organ, mixed stages Floral organ, mixed stages Floral organ, mixed stages Floral organ, mixed stages
*BrARF5*-*3*	JN979467	1,413	470	52.6	7.39	A6	7.92		
*BrARF6*	JN979468	2,499	832	91.7	6.52	A8	5.25	EX023086 EX073902^d^ EX073291^d^ EX117497	Whole plant, cold treated Cotyledon, greening stage Root, 1 month old Root, 1 month old
*BrARF7*-*1*	JN979469	4,425	1,474	163.2	7.15	A10	7.30		
*BrARF7*-*2*	JN979470	3,303	1,100	121.2	6.64	A2	5.15		
*BrARF8*-*1*	JN979471	2,343	780	87.3	6.21	A7	16.70	EX079023 EX060038 EX117964 KFFO-062B05	Silique Whole plant, salt treated Cotyledon, greening stage Floral organ, mixed stages
*BrARF8*-*2*	JN979472	2,535	844	93.1	6.31	A4	6.05	EX126938	Non-photosynthetic mature leaf
*BrARF9*-*1*	JN979473	1,809	602	68.3	6.51	A1	7.57	EX099543 EX101519	Root, 1, 3, and 7 weeks old Root, 1, 3, and 7 weeks old
*BrARF9*-*2*	JN979474	1,890	629	71.3	6.49	A3	6.82	EX100066 EX136378	Root, 1, 3, and 7 weeks old Root, mixed stage and treatment
*BrARF10*	JN979475	2,118	705	77.9	7.42	A7	10.72	EX044295 EX135102	Floral bud, >2 mm in size Root, mixed stage and treatment
*BrARF11*	JN979476	1,755	584	65.9	7.16	A5	23.31		
*BrARF16*-*1*	JN979477	1,944	647	71.5	7.96	A1	3.59		
*BrARF16*-*2*	JN979478	2,085	694	77.3	7.66	A3	4.67		
*BrARF17*-*1*	JN979479	1,641	546	59.9	5.16	A7	14.07	EX057163	Whole plant, salt treated
*BrARF17*-*2*	JN979480	1,641	546	59.9	5.55	A7	21.27		
*BrARF18*-*1*	JN979481	3,168	1,055	118.8	8.05	A9	6.74	EX054017 EX057541 EX072589 EX074532 EX062740	Floral bud, <2 mm in size Whole plant, salt treated Root, 1 month old Root, 1 month old Non-photosynthetic mature leaf
*BrARF18*-*2*	JN979482	1,668	555	62.4	6.24	A4	0.52		
*BrARF19*-*1*	JN979483	3,063	1,020	112.6	6.71	A8	2.78	EX060157 EX060933	Whole plant, salt treated Whole plant, salt treated
*BrARF19*-*2*	JN979484	3,150	1,049	116.1	6.51	A6	7.47	EX017752 EX023291	Whole plant, cold treated Whole plant, cold treated
*BrARF24*	JN979485	1,623	540	61.3	6.24	A9	22.99		
*BrARF25*	JN979486	1,650	549	62.4	6.30	A9	27.14		
*BrARF26*	JN979487	1,668	555	62.8	6.61	A6	19.37		
*BrARF27*	JN979488	1,626	541	61.9	6.44	A1	25.57		

^a^Molecular weight, ^b^Isoelectric point, ^c^Chromosome, ^d^Alternative splicing form

### Phylogenetic relationship of the *BrARF* gene family

**Fig. 2:**
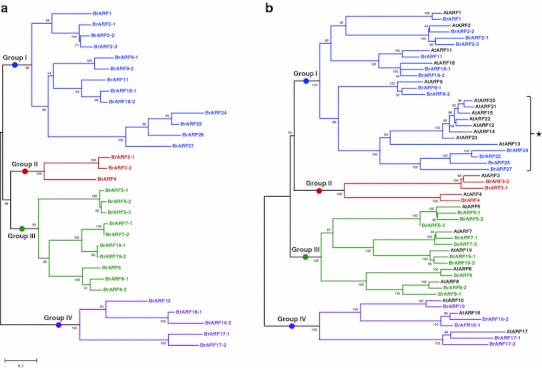
Phylogenetic tree of *B. rapa* and *A. thaliana* *ARF* genes. Phylogenetic trees of 31 *BrARF* genes alone (**a**) and together with 23 *AtARF* genes (**b**) were generated using MEGA5 program by neighbor-joining analysis. Bootstrap values (above 50 %) are indicated on the branches and the branch length reflects the estimated number of substitutions per 100 sites. Genes in the same group are represented by color bars. A lineage-specific local cluster of the Group I genes is indicated with an *asterisk*

A phylogenetic analysis of 31 *BrARF* sequences was performed to classify subgroups and to identify relationships between genes. Both neighbor-joining and maximum likelihood algorithms produced similar trees (Supplementary Figs. 1, 2), and the phylogenetic patterns of the subgroups in both trees were consistent with the previously reported trees using *A. thaliana* and rice ARF proteins (Okushima et al. 2005; Wang et al. 2007). Figure 2a shows a phylogeny of *BrARF* genes based on the neighbor-joining method, indicating that the genes fall into four subgroups: Group I (*BrARF1*, *2*-*1*, *2*-*2*, *2*-*3*, *9*-*1*, *9*-*2*, *11*, *18*-*1*, *18*-*2*, *24*, *25*, *26*, *27*), II (*BrARF 3*-*1*, *3*-*2*, *4*), III (*BrARF* *5*-*1*, *5*-*2*, *5*-*3*, *6*, *7*-*1*, *7*-*2*, *8*-*1*, *8*-*2*, *19*-*1*, *19*-*2*), and IV (*BrARF10*, *16*-*1*, *16*-*2*, *17*-*1*, *17*-*2*). A composite tree for the 23 *AtARF* (Okushima et al. 2005) and 31 *BrARF* genes was consistent with that of the *BrARF* gene tree (Fig. 2b). All *BrARF* subgroups present in the *BrARF* gene tree were present in the composite phylogenetic tree for both genes, which support the categorization of *BrARF* genes into four subgroups. In addition, the tree indicates homologous relationships between the genes of *B. rapa* and *A. thaliana*. Most of the *AtARF* genes have one to three homologous counterparts of the *BrARFs*, and the exon–intron structures of the genes in the same subgroup were similar (Fig. 1). The phylogenetic distribution of *A. thaliana* and *B. rapa* *ARF* genes detected a local clustering of particular types. Four genes of Group I (*BrARF24*–*27*) formed a local cluster separated from the *AtARF12*–*15* and *20*–*23* subclade. Unlike the *Arabidopsis* genes that are tandem clustered in the upper arm of *A. thaliana* chromosome 1 and appear to be specifically expressed in the seed and embryo (Okushima et al. 2005), these genes might have been generated by dispersed duplication of *BrARF27* recently after the divergence of *A. thaliana* and *B*. *rapa*, followed by negative selection based on the location of genes on the *B. rapa* chromosomes and *K*_s_–*K*_a_ comparison between the paralogous gene pairs (Table 1, Supplementary Tables 1 and 2). In addition, they are not expressed in any of the tissues investigated.

**Table 2:** Homologous counterpart pairs of *A. thaliana* and *B. rapa ARF* genes

Group	Gene	*A. thaliana*	*AK*	*B. rapa*	*AK*	*K*_s_
I	ARF1	*AtARF1*	D	*BrARF1*	D	0.31
	ARF2	*AtARF2*	X	*BrARF2*-*1*	X	0.47
				*BrARF2*-*2*	X	0.49
				*BrARF2*-*3*	X	0.47
	ARF9	*AtARF9*	U	*BrARF9*-*1*	U	0.37
				*BrARF9*-*2*	U	0.36
	ARF11	*AtARF11*	J	*BrARF11*	J	0.39
	ARF12	*AtARF12*	B			
	ARF13	*AtARF13*	B			
	ARF14	*AtARF14*	B			
	ARF15	*AtARF15*	B			
	ARF18	*AtARF18*	N	*BrARF18*-*1*	N	0.39
				*BrARF18*-*2*	N	0.35
	ARF20	*AtARF20*	B			
	ARF21	*AtARF21*	B			
	ARF22	*AtARF22*	B			
	ARF23	*AtARF23*	C			
				*BrARF24*	K	
				*BrARF25*	D	
				*BrARF26*	M	
				*BrARF27*	L	
II	ARF3	*AtARF3*	J	*BrARF3*-*1*	J	0.38
				*BrARF3*-*2*	J	0.40
	ARF4	*AtARF4*	W-X	*BrARF4*	W-X	0.35
III	ARF5	*AtARF5*	B	*BrARF5*-*1*	B	0.51
				*BrARF5*-*2*	A	0.46
				*BrARF5*-*3*	A	0.42
	ARF6	*AtARF6*	B	*BrARF6*	B	0.37
	ARF7	*AtARF7*	R	*BrARF7*-*1*	R	0.35
				*BrARF7*-*2*	R	0.40
	ARF8	*AtARF8*	S	*BrARF8*-*1*	E	0.69
				*BrARF8*-*2*	S	0.34
	ARF19	*AtARF19*	A	*BrARF19*-*1*	A	0.39
				*BrARF19*-*2*	A	0.44
IV	ARF10	*AtARF10*	I	*BrARF10*	I	0.55
	ARF16	*AtARF16*	U	*BrARF16*-*1*	U	0.68
				*BrARF16*-*2*	U	0.90
	ARF17	*AtARF17*	E	*BrARF17*-*1*	E	0.77
				*BrARF17*-*2*	E	1.04

Genomic position mapped on the ancestral karyotype genome building block (*AK*) and synonymous substitution rate (*K*_s_) between the homologous gene pair are presented

**Fig. 3:**
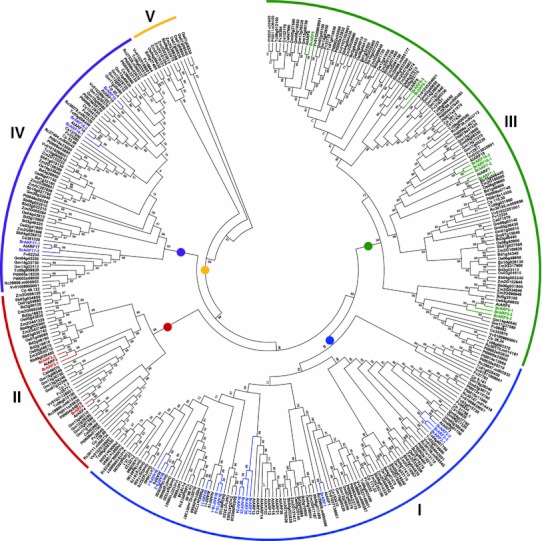
Phylogenetic relationships of plant *ARF* gene family. The neighbor-joining tree was constructed using *31* *B. rapa*, *23* *A. thaliana*, *13* papaya, *16* cucumber, *17* strawberry, *54* soybean, *36* poplar, *18* castor bean, *19* cacao, *19* grape, *24* *B. distachyon*, *28* rice, *25* sorghum, and *37* maize putative *ARF* genes. Groups of genes are represented by *color arcs*. Bootstrap values (above 50 %) are indicated on the branches and the branch length reflects the estimated number of substitution per 100 sites. *BrARFs* and *AtARFs* were color coded according to their subgroup in Fig. 2. *At* *Arabidopsis thaliana*, *Br* *Brassica rapa*, *Cp* *Carica papaya*, *Cs* *Cucumis sativus*, *Fv* *Fragaria vesca*, *Gm* *Glycine max*, *Pt* *Populus trichocarpa*, *Rc* *Ricinus communis*, *Tc* *Theobroma cacao*, *Vv* *Vitis vinifera*, *Bd* *Brachypodium distachyon*, *Os* *Oryza sativa*, *Sb* *Sorghum bicolor*, *Zm* *Zea mays*

Further phylogenetic reconstruction using *ARF* genes from the sequenced plant genomes was performed to assess the support for the *ARF* gene subgroups and to investigate the evolution of *BrARF* genes (Fig. 3). A phylogenetic tree constructed using the protein sequences of the *ARF* genes from the sequenced plant genomes shows that plant *ARF* genes are divided into five major clades. All the dicot genes, including *BrARFs* and *AtARFs,* were clustered into four subgroups (Group I–IV) that show a similar tree topology to the composite tree for *AtARF* and *BrARF* genes in Fig. 2b. Interestingly, Group V contains only monocot genes, suggesting that this group arose in monocots or was lost from dicots after the divergence of monocots and dicots. Taken together, these results suggest that *BrARF* genes shared the same origin with the *AtARF* genes, and they were more amplified after speciation from *A. thaliana*, presumably by WGT.

### Evolutionary origin and divergence of *BrARF* genes

**Fig. 4:**
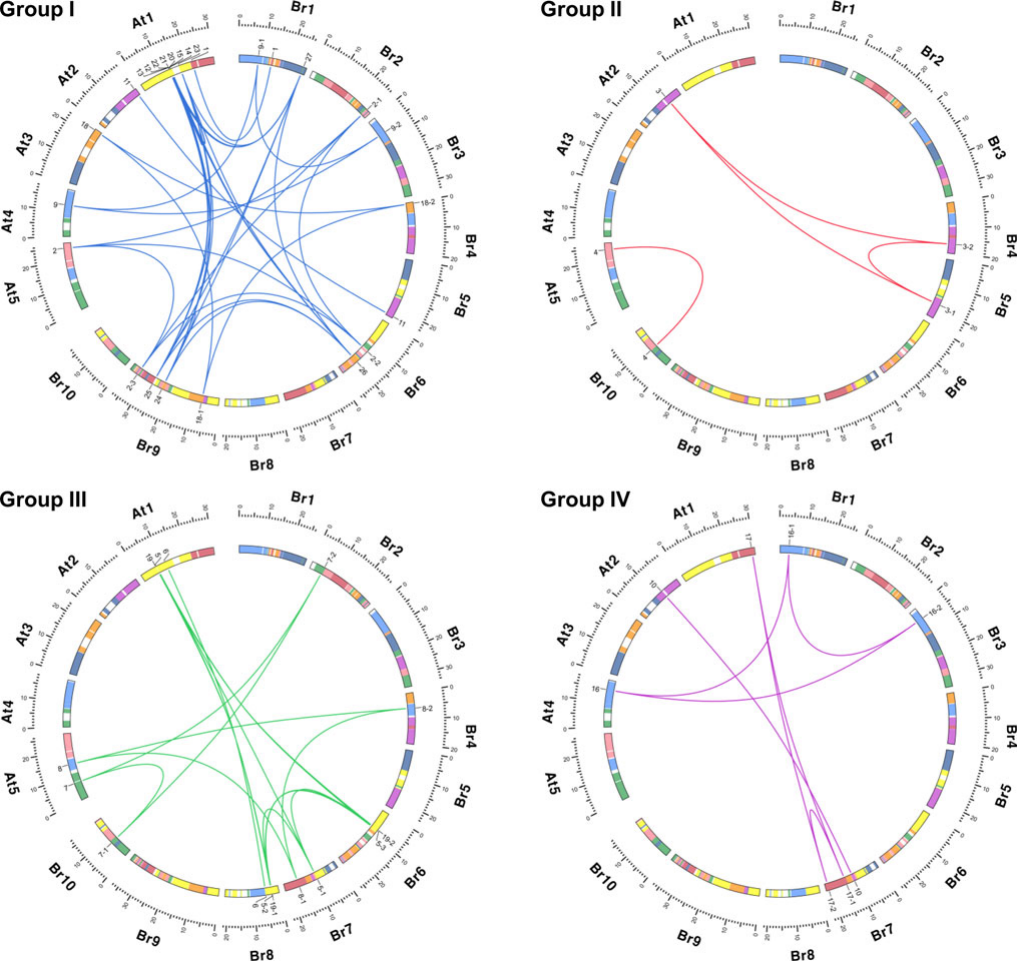
Circos diagrams of *ARF* gene pairs. *BrARF* genes in each group are plotted against their homologous *AtARF* counterparts. Individual chromosomes of *B. rapa* (Br) and *A. thaliana* (At) are shown as ancestral karyotype genome building blocks to represent the shared ancestral origin of their genomes

A genome-level comparison between the *B. rapa* and *A. thaliana* chromosomes presented an insight into the origin and evolution of the *BrARF* gene family. Considering the recent WGT event, the *B. rapa* genome is expected to have approximately 60 *ARF* genes. However, we could identify only 31 *BrARF* genes in the genome, suggesting the loss of redundant genes through the diploidization process after WGT. To determine the evolutionary origin of *BrARF* genes, we compared their genomic positions with those of their *A. thaliana* counterparts with the view of 24 *AK* genome building blocks. A synteny block comparison between homologous gene pairs revealed that all *AtARF* genes, with the exception of the tandem duplicated genes on chromosome 1 (*AtARF12*–*15*, *20*–*23*), have their *BrARF* homologs on the same *AK* genome building blocks (Table 2; Fig. 4). For example, *AtARF2* in Group I has their three homologs on the triplicated X blocks of the *B. rapa* chromosome. Only *AtARF5* and *AtARF8* have additional homologs on the distinct *AK* blocks of *B. rapa* genome suggesting genomic rearrangement. Furthermore, most of the *BrARF* genes, except the *BrARF2* and *5* genes, which have triplicated paralogs, were duplicates or single members, suggesting a loss of paralogs throughout the genome.

**Fig. 5:**
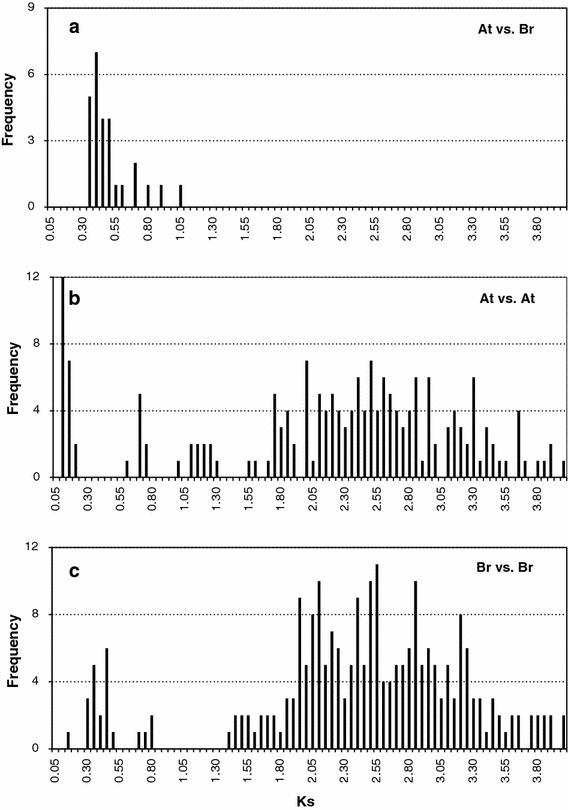
Age distribution of *ARF* genes in the genome of *A. thaliana* (At) and *B. rapa* (Br) viewed through the frequency distribution of relative *K*_s_ modes. Distributions of *K*_s_ values were obtained from orthologous gene sets (**a**) between the genomes and paralogous genes sets (**b**, **c**) in the *A. thaliana* and *B. rapa* genomes. The *vertical axes* indicate the frequency of paired sequences and the *horizontal axes* denote the *K*_s_ values at 0.05 intervals. The *black bars* depict the positions of the modes of the *K*_s_ distribution obtained from orthologous or paralogous gene pairs

The age distributions of the orthologous pairs between the *AtARF* and *BrARF* genes displayed a single major peak at *K*_s_ = 0.35–0.40, indicating that the *ARF* genes of the two genomes were separated approximately 13 Mya (Fig. 5a). This is in good agreement with our previous study of speciation between the *Brassica* and *Arabidopsis* genomes (Mun et al. 2009a). A comparison of the *K*_s_ mode for the paralogs in both genomes identified overall similar *K*_s_ distribution patterns above *K*_s_ = 1.3 in both genomes, representing the origin of the genes from ancient polyploidy events. However, distinct peaks for each genome were found at *K*_s_ = 0.05–0.10 for *AtARFs* and *K*_s_ = 0.25–0.35 for *BrARFs*, suggesting the occurrence of additional members for each genome after speciation (Fig. 5b, c). In particular, note that a peak at *K*_s_ = 0.30–0.40 for *B. rapa* is responsible for the *K*_s_ between the paralog pairs generated by WGT, whereas a peak for *A. thaliana* paralogs at *K*_s_ = 0.05–0.10 is responsible for *K*_s_ between the tandem clusters of *AtARF12*–*15* and *20*–*23* (Supplementary Tables 2 and 3). Interestingly, duplicated or triplicated *BrARFs* had higher *K*_s_ values for their *AtARF* orthologs than those of single-member *BrARFs* in a two-tailed* t* test (*P* < 0.05), indicating a higher sequence divergence of multiplicated members of the *BrARF* genes (Table 2). This finding proposes a hypothesis that *BrARF* genes, which shared the same origin from the ancestral genome blocks with *AtARF* genes, were amplified by WGT, but the redundant genes might have been lost during genome diploidization due to events such as the accumulation of mutations (sequence divergence), loss of redundant genes, recombination, or epigenetic marking.

**Fig. 6:**
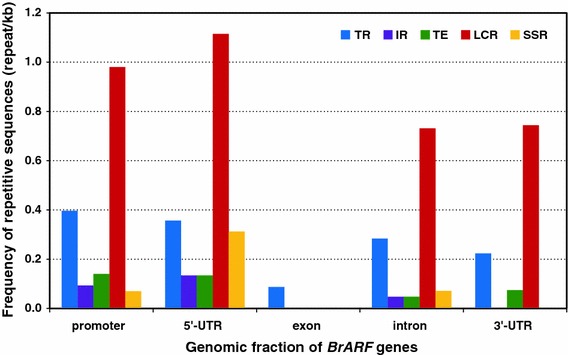
Frequency distribution of five repetitive sequences in the genomic fraction of *BrARF* genes. The number of repetitive sequences was counted per every 1 kb of promoter, 5′-UTR, exon, intron, and 3′-UTR of *BrARFs*. *TR* tandem repeat, *IR* inverted repeat, *TE* transposable element, *LCR* low complexity repeat, *SSR* simple sequence repeat

To characterize the sequence divergence of the genes, the spatial distribution of repetitive sequences with respect to the genomic position of *BrARF* genes was examined. We investigated the distribution of five types of repetitive sequences frequently found in the promoter and coding regions of the *B. rapa* genome (TR, IR, TE, LCR, and SSR). To correlate repetitive sequences with specific genomic fractions, we assigned five categories of sequences, namely, (1) promoter, (2) 5′-UTR, (3) coding exon, (4) intron, and (5) 3′-UTR. As shown in Fig. 6, all the *BrARF* genes had repetitive sequence insertions and the repetitive sequences were frequent both in the promoter and 5′-UTR regions (1.7 and 2.1 repeats/kb, respectively) compared with the exons (0.1 repeats/kb), introns (1.2 repeats/kb), and 3′-UTR regions (1.0 repeats/kb), due primarily to a higher frequency of LCR. In addition, repeat frequency varied between the different transcribed fractions. Most of the repetitive sequences in the transcribed regions were detected in 5′-UTRs, introns, and 3′-UTRs, with the highest repeat frequency in the 5′-UTRs, which were characterized by elevated levels of LCR and SSR. Exons were significantly underestimated in all repetitive sequences with the exception of TR. Interestingly, even in the duplicate or triplicate paralogs, the degree of repetitive sequence insertion is quite different between paralog members, suggesting that sequence divergence occurred through the insertion of repetitive sequences, mainly in the promoter or noncoding regions of the transcribed fraction. The frequent occurrence of repetitive sequence insertion, both in the promoter and transcribed regions of the genes, may promote the divergence of triplicated genes, resulting in more variants. This finding suggests that the alteration of sequences in the promoter and noncoding regions of the triplicated genes leads to the divergence of expression patterns and function or gene silencing to the pseudogene.

### RNA-Seq analysis of *BrARF* gene expression

To determine the expressional characteristics of each *BrARF* gene member according to auxin treatment and developmental process of the flower, we investigated the expression pattern of the genes using next-generation RNA-Seq technology. The recent development of the RNA-Seq method provides not only information on genome-wide gene expression, but also has the advantage of higher sensitivity and a greater dynamic range of gene expression than array-based technologies (Severin et al. 2010). The sequencing of seedlings, anther, and pistil transcriptomes generated 100 bp paired-end reads for each library covering at least 17× of the genome. The percentage of reads aligned to the genome averaged around 74–75 %, which represents both the quality of the libraries and the relative completeness of the *B. rapa* draft genome. The current draft genome sequence of *B. rapa* (284 Mb) covers only 54 % of the whole estimated genome (529 Mb) (Johnston et al. 2005). Through the comparative analysis of sequence datasets against the *B. rapa* draft genome, a total of 35,220 genes in seedlings, 37,565 genes in anthers, and 35,477 genes in pistils were expressed (data not shown). From these results, we extracted the data for the *BrARF* gene family and conducted further analysis.

**Fig. 7:**
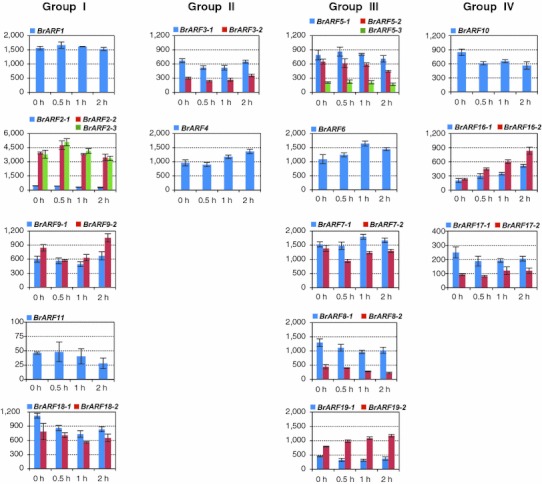
Expression of *BrARF* genes in response to exogenous auxin treatment. Five-day-old seedlings were harvested after 0, 0.5, 1, and 2 h of incubation in 0.5× MS agar media containing 1 μM NAA. The whole mRNA level for each gene per treatment was estimated using mRNA sequencing followed by TMM normalizaton. The average value of three biological replicates was presented with the standard deviation. No expression of *BrARF24*–*27* was detected in this experiment. *X* axes indicate the NAA incubation time and *Y* axes denote normalized read count

It has been reported that auxin treatment induced or repressed the expression of some *ARF* members in *A. thaliana* (Okushima et al. 2005), rice (Wang et al. 2007), and maize (Xing et al. 2011). The response of *BrARF* genes to exogenous auxin stimuli was investigated in seedlings after 1 μM NAA treatment for 2 h. RNA-Seq analysis revealed that a total of 27 out of 31 *BrARFs* displayed transcriptional activity in seedlings, and no expression was detected for *BrARF24*–*27* under any of the conditions tested in this study (Fig. 7). The most highly expressed genes in seedlings was *BrARF2*-*2* and *2*-*3*, which showed 3- to 85-fold higher expression than other members. Most of the *BrARFs* were responsive to exogenous auxin treatment but showed diverse expression patterns. As with their *Arabidopsis* homologs *AtARF16* and *19* (Okushima et al. 2005; Wang et al. 2005), auxin treatment gradually enhanced transcription for *BrARF16*-*1*, *16*-*2*, and *19*-*2*, while decreased expression was found for *BrARF2*-*1*, *5*-*2*, *8*-*2*, and *10*. In addition, 8 genes showed up-regulation by auxin treatment followed by down-regulation (*BrARF1*, *2*-*2*, *2*-*3*, *5*-*1*, *5*-*3*, *6*, *7*-*1*, and *11*) and 12 genes were down-regulated first but up-regulated later (*BrARF3*-*1*, *3*-*2*, *4*, *7*-*2*, *8*-*1*, *9*-*1*, *9*-*2*, *17*-*1*, *17*-*2*, *18*-*1*, *18*-*2*, and *19*-*1*). In either cases, transcriptional changes were observed within 0.5–1 h after auxin treatment. Of particular interest, one of the duplicate or triplicate paralog members was more abundantly expressed than the other members, suggesting that there are functional redundancies among the genes. Furthermore, expression patterns between paralogs in response to auxin treatment were different. These transcriptional differences may involve the modification of regulatory elements. A database search of plant promoters (PLACE) detected several auxin response elements in the promoter region of all of *BrARF*s except *BrARF19*-*1*, supporting the auxin responsiveness of *BrARF* genes (Supplementary Table 4). This finding demonstrates the complexity of the auxin-regulated expression of *BrARF* genes and thus the relationship between auxin response elements and the change in expression of *BrARF* genes by auxin treatment need to be further investigated.

**Fig. 8:**
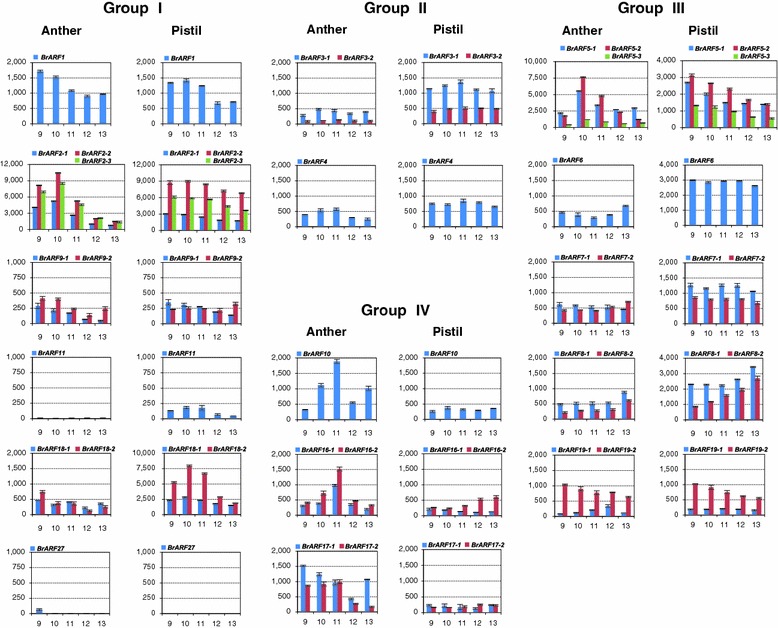
Expression profiling of *BrARF* genes during anther and pistil development. Anther and pistil at floral development stages 9–13 were harvested from growth chamber-grown plants. Whole mRNA levels for each gene in the anther and pistil tissues were estimated using RNA sequencing followed by TMM normalization. The average value of three biological replicates was presented with the standard deviation. No expression of *BrARF24–26* was detected in the anther or pistil tissues at any stage. *X* axes indicate flower development stage and *Y* axes denote normalized read count

We also studied the expression profile of *BrARF* genes during flower development based on the recent functional studies of *AtARF* genes that showed their important role in flower, embryo, and seed development (Hardtke and Berleth 1998; Schruff et al. 2005; Nishimura et al. 2005; Finet et al. 2010). An investigation of gene expression was performed with anthers and pistils at floral development stages 9–13, at which both reproductive organs rapidly elongate and gametophyte development is completed (Smyth et al. 1990; Yu et al. 2005). It is known that *ARF* genes are constitutively expressed in plant tissues (Wang et al. 2007). However, RNA-Seq analysis of *BrARF* genes exhibited dynamic expression patterns in reproductive organs during flower development. Except *BrARF11*, *24*, *25*, *26*, and *27* in Group I, all the *BrARF* genes were expressed in both the anther and pistil tissues, and some of them showed relatively higher expression in specific tissues and development stages (Fig. 8). In addition, the expression patterns of duplicate or triplicate paralogs also varied considerably. Similar to auxin treatment, one of the duplicates or triplicates was dominantly expressed over the others. Genes in Group II were more abundantly expressed in the pistil than the anther, whereas Group IV members showed relatively high expression in anther. Genes in Groups I and III, however, presented complicated expression patterns in that some genes exhibited relatively high expression in the pistil, whereas other genes demonstrated significant induction at specific stages of anther and pistil development. For example *BrARF2*-*2* and *2*-*3* in Group I and *BrARF5*-*1* and *5*-*2* in Group III were highly induced at stage 10 of anther development (uni-nuclear stage of male gametophyte), whereas *BrARF18*-*2* in Group I and *BrARF5*-*1* and *5*-*2* in Group III showed up-regulation in pistils at the floral development stage 9–10 (initiation of ovule primodia). Among the genes, the most predominantly expressed gene in both the anther and pistil was *BrARF2*-*2*, and this is in good agreement with the presence of abundant ESTs (Table 1) and expression data in seedlings (Fig. 7). It is worthy to note that this gene might be the primary auxin response gene in *B. rapa* because its mRNA was highly up-regulated when treated with exogenous auxin and significantly induced or sustained at high level at specific developmental stages of the anther or pistil. Transcriptional differentiation of paralog members was also noticed between vegetative and floral tissues. In seedlings, *BrARF5*-*1* and *18*-*1* were abundantly expressed, whereas *BrARF5*-*2* and *18*-*2* were dominant forms expressed in floral tissues. In addition, consistent with the results shown in Fig. 7, *BrARF24*, *25*, and *26* were not expressed in anthers or pistils at any development stages. Only *BrARF27* showed very low expression levels at the early stage of anther development, suggesting that genes emerging recently by dispersed duplication are more likely to be silenced. Otherwise, their function can be restricted to a specific tissue or development stage including embryogenesis or seed development based on the role of *A. thaliana* homologs (Okushima et al. 2005).

## Discussion

Polyploidy is ubiquitous in the plant genomes (Cui et al. 2006; De Bodt et al. 2005; Jiao et al. 2011). All the plant genomes characterized so far experienced whole genome duplication events during their evolution. Polyploidy facilitates the accumulation of duplicated genes in the genome, leading to the increased complexity and diversity of the gene network. Duplication at the whole genome level led to gene redundancy, and resulting redundant copies of amplified genes might be pseudogenes (nonfunction) or gain additional (subfunction) or novel functions (neofunction). Otherwise, the duplicated genes may play a role in preventing potential harmful mutations (buffering). In any of these cases, the ultimate fate of duplicated genes varied according to the individual plant and species (Sémon and Wolfe 2007; Taylor and Raes 2004). In this study, we investigated the organization and expression of 31 *BrARF* genes. Phylogenetic analysis and comparative mapping of genes on the *AK* genome blocks revealed that the *BrARF* gene family consists of four sister groups. The tree topology of the current analysis is very similar to the previously reported trees based on *A. thaliana* and rice ARF proteins (Okushima et al. 2005; Wang et al. 2007). Furthermore, most of the *BrARF* genes shared same ancestral origin with the *AtARF* homologs mapped to the same *AK* blocks. Thus, the expansion of the *BrARF* gene family could be explained by the recent WGT (Mun et al. 2009a; The *Brassica* *rapa* Genome Sequencing Project Consortium 2011). Considering that *B. rapa* underwent WGT, the *BrARF* gene family ought to have approximately three times as many members as that of *A. thaliana*. However, only 31 members were identified in the *B. rapa* genome, suggesting a substantial loss of genes following hexaploidy formation by WGT.

The collapse of the *BrARF* gene complement might be a result of genome-level gene loss, termed paralog reduction or fractionation, which is typical of the diploidization of eukaryotes. It is noteworthy that, following polyploidy events, loss events of gene duplicates through processes such as epigenetic silencing, pseudogenization, and deletion of chromosomal segments containing one or more genes following polyploidy randomly occurred in the genome (Sankoff et al. 2010). The current analysis of *ARF* gene distribution in *B. rapa* agrees with this hypothesis, where loss of paralog members had occurred in the genome. Although it is uncertain as to how the redundant genes were deleted, it is possible that the insertion of repetitive sequences might lead to the pseudogenization of paralogs. Analysis of repetitive sequences in the promoter and non-coding regions in the genic region of *BrARF* genes revealed that all the *BrARF* genes contain repetitive sequence insertions in the sequences. Moreover, the higher frequency of repetitive sequences, especially LCR, which is the most abundant repetitive sequence in the *B. rapa* genome, in the promoter, 5′-UTR, and intronic regions was identified, suggesting that sequence divergence may possibly be preceded by the suppression of transcription and/or a change to the exonic splicing pattern. Repetitive sequence insertion possibly changes the structure of the original gene. For example, transposon activation can induce the alteration of cis-regulatory elements or sequence loss. In addition, LCR and SSR in duplicate genes may become active to increase the mutation rate when the species suffer under harsh environments. TR and IR sequences together promote the variation and divergence of the introns of paralogs. This finding is consistent with recent reports that the splicing pattern and cis-regulatory region of the duplicates genes changed rapidly after duplication and led to changes in the protein constitutions (Chen 2010; Paterson et al. 2009; Zhang et al. 2009). Thus, the variation in the promoter and genic regions may possibly promote sequence divergence and gene inactivation and eventually result in paralog reduction in the *BrARF* gene family. We note that the *BrARF24*, *25*, and *26* genes, which may have originated from the dispersed duplication of *BrARF27* after WGT, showed no expression in the tissues investigated. This finding indicates that the genes recently emerging after WGT were silenced or gained different expression characteristics compared with other members, suggesting the birth and death of duplicated paralogs is an ongoing process. Therefore, internal sequence divergence along with natural selection can serve at the ultimate factors that influence the fate of duplicated genes.

The dynamic and differential distribution of auxin hormone in the plant controls the diverse morphological differentiation, development, and growth. Under domestication and selection, *Brassica* species have a notable propensity to develop new morphological variants rapidly (Paterson et al. 2001). The current draft genome showed that the gene families involved in auxin signal transduction (transport inhibitor response 1, *TIR1*; topless, *TPL*; *ARF*; *AUX/IAA*) have been expanded and amplification by tandem duplication beyond that of *A. thaliana* can be observed for *GH3* and *SAUR* in the *B. rapa* genome (The *Brassica* *rapa* Genome Sequencing Project Consortium 2011). *ARFs* are transcription factors that are upstream regulators of the auxin-related signaling process. Although several members were deleted and silenced after WGT, the expression of duplicated *ARF* genes in *B. rapa* would amplify expression of downstream auxin-related genes in the signaling pathway. Moreover, the divergence of paralogs may result in the alteration of the function and expression pattern of an ancestral gene. Of practical importance, functional changes to *BrARF* genes may have crucial effect on gametophyte and seed development in *B. rapa*, considering the role of *AtARF* genes during flower development. Since a limited number of ESTs was reported, we studied the expression of all *BrARF* genes in seedlings and flowers by whole transcriptome sequencing to investigate their global expression pattern and putative function. The RNA-Seq showed a great dynamic range of gene expression, with low variation between technical replicates.

In this study, we found that 27 *BrARF* genes were expressed in seedlings and that they responded to exogenous auxin treatment. Identification of various auxin responsive elements in the promoter region seems to be related with a transcriptional change to the *BrARF* genes. We found that the expression of *BrARF16*-*1*, *16*-*2*, and *19*-*2* was increased, whereas that of *BrARF2*-*1*, *5*-*2*, *8*-*2*, and *10* was decreased by auxin treatment, although they contained the same auxin responsive transcriptional activation element in the promoter region. Posttranscriptional down-regulation of the transcript by miRNA is possible for these genes, based on action of miRNAs on *AtARF* transcripts (Wang et al. 2005; Wu et al. 2006; Yang et al. 2006). *BrARF2*-*1* and *5*-*2* have the target sites for miRNA396 and miRNA156, respectively. *BrARF10* contains those for miRNA156 and miRNA160; however, the target sites of miRNA160 were also detected in *BrARF16*-*1* and *16*-*2*. The differential insertion of repetitive sequences in the promoter region or unidentified novel miRNA of additional epigenetic markings may be involved in the regulation of gene expression. Therefore, we anticipate that the regulation of *BrARF* gene expression in auxin signaling is more complicated. Expression changes between gene duplicates in polyploidal *B. rapa* may reflect the retention of redundant copies with functional divergence. The expression patterns of *BrARF* genes in the anther and pistil also indicate that several members are transcriptionally induced at specific stages of anther or pistil development. High expression levels of *BrARF2*-*2*, *2*-*3*, *5*-*1*, and *5*-*2* at stage 9–10 in both anther and pistil development suggest that these genes play a more general role during early flower development. In particular, the differential expression of *BrARF10*, *16*-*1*, *16*-*2*, *17*-*1*, and *17*-*2* in the anther and *BrARF3*-*1*, *6*, *8*-*1*, *8*-*2*, and *18*-*1* in the pistil implies that these genes might be involved in a distinct floral development process. This result indicates that more diverse functions can be played by *BrARF* in floral tissues. Although the role of *BrARF* genes needs to be further investigated, the information on the structure and expression of *BrARF* generated here will accelerate their functional analysis in the future.

The current work has contributed to an increased knowledge of the *ARF* gene family in *B. rapa*. Based on the genomic organization and expression characteristics, we can hypothesize that overrepresentation of *BrARF* genes, together with other auxin-related genes in the signaling pathway, may enrich the auxin-related gene networks in the plant. Considering that the possible role of multiple auxin-related gene networks in environmental adaptation is complex, systematic studies on the target genes of *ARF* in auxin signaling pathway, such as *AUX/IAA*, *GH3*, and *SAUR*, will increase our understanding of the development and adaptation of *B. rapa*.

## Electronic supplementary material

Below is the link to the electronic supplementary material.

Supplementary material 1 (PPTX 122 kb)

Supplementary material 2 (DOCX 24 kb)
