# Supplementary material for: Auxin response factor gene family in Brassica rapa: genomic organization, divergence, expression, and evolution
Source: Mol Genet Genomics. 2012 Aug 24;287(10):765–84. doi: 10.1007/s00438-012-0718-4 (PMC3459075; doi:10.1007/s00438-012-0718-4)
Supplement: Supplementary file 1 — Supplementary material 1 (PPTX 122 kb) [file 438_2012_718_MOESM1_ESM.pptx]

## Slide 1
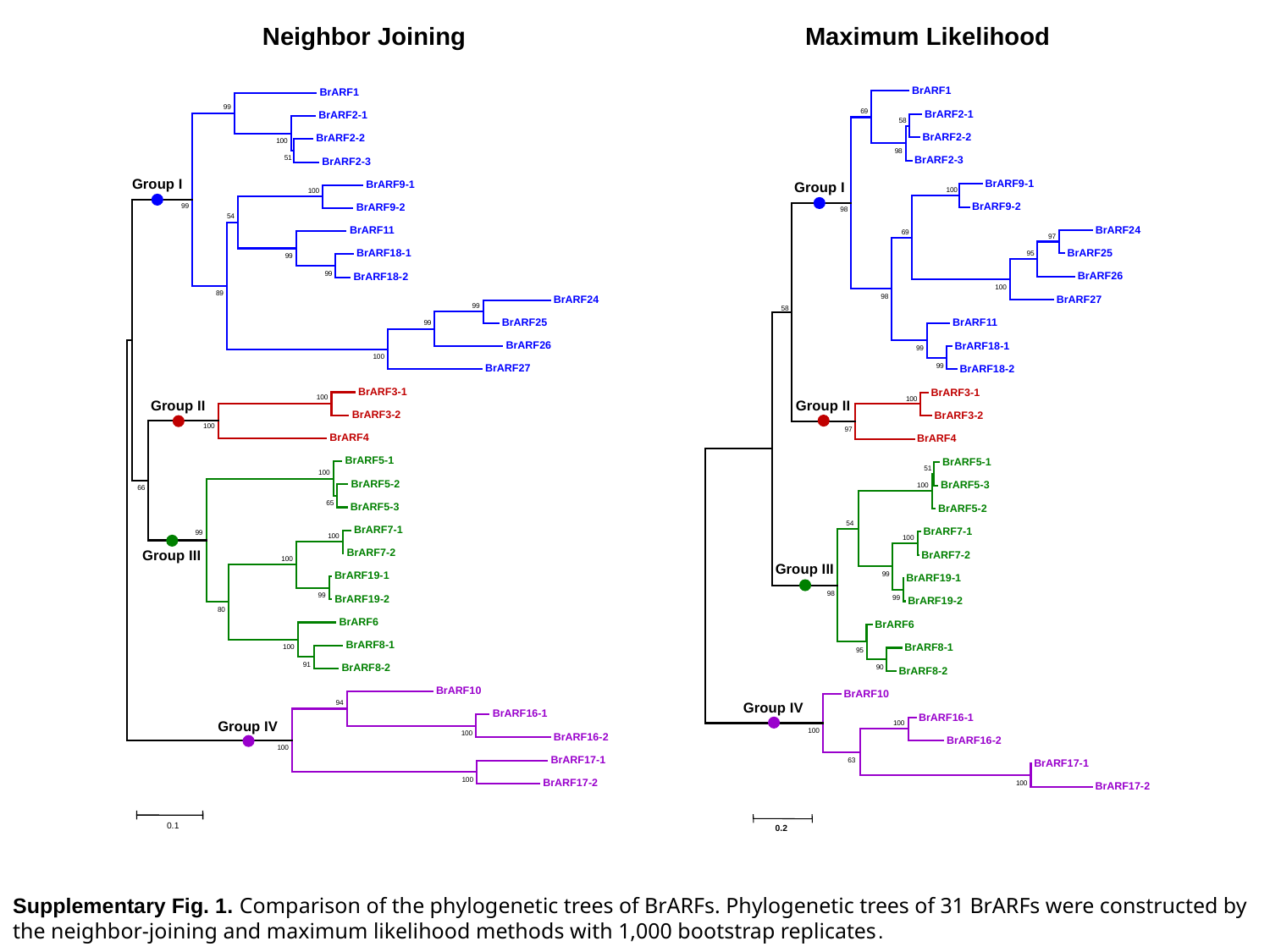

Neighbor Joining
 BrARF1
99
 BrARF2-1
 BrARF2-2
100
51
 BrARF2-3
Group I
 BrARF9-1
100
 BrARF9-2
99
54
 BrARF11
 BrARF18-1
99
99
 BrARF18-2
89
 BrARF24
99
 BrARF25
99
 BrARF26
100
 BrARF27
 BrARF3-1
Group II
100
 BrARF3-2
100
 BrARF4
 BrARF5-1
100
 BrARF5-2
66
65
 BrARF5-3
 BrARF7-1
99
100
Group III
 BrARF7-2
100
 BrARF19-1
99
 BrARF19-2
80
 BrARF6
 BrARF8-1
100
91
 BrARF8-2
 BrARF10
94
 BrARF16-1
Group IV
100
 BrARF16-2
100
 BrARF17-1
100
 BrARF17-2
0.1
Maximum Likelihood
BrARF1
69
BrARF2-1
58
BrARF2-2
98
BrARF2-3
Group I
BrARF9-1
100
BrARF9-2
98
BrARF24
69
97
BrARF25
95
BrARF26
100
98
BrARF27
58
BrARF11
BrARF18-1
99
99
BrARF18-2
BrARF3-1
Group II
100
BrARF3-2
97
BrARF4
BrARF5-1
51
BrARF5-3
100
BrARF5-2
54
BrARF7-1
100
BrARF7-2
Group III
99
BrARF19-1
98
99
BrARF19-2
BrARF6
BrARF8-1
95
90
BrARF8-2
BrARF10
Group IV
BrARF16-1
100
100
BrARF16-2
63
BrARF17-1
100
BrARF17-2
0.2
Supplementary Fig. 1. Comparison of the phylogenetic trees of BrARFs. Phylogenetic trees of 31 BrARFs were constructed by the neighbor-joining and maximum likelihood methods with 1,000 bootstrap replicates.

## Slide 2
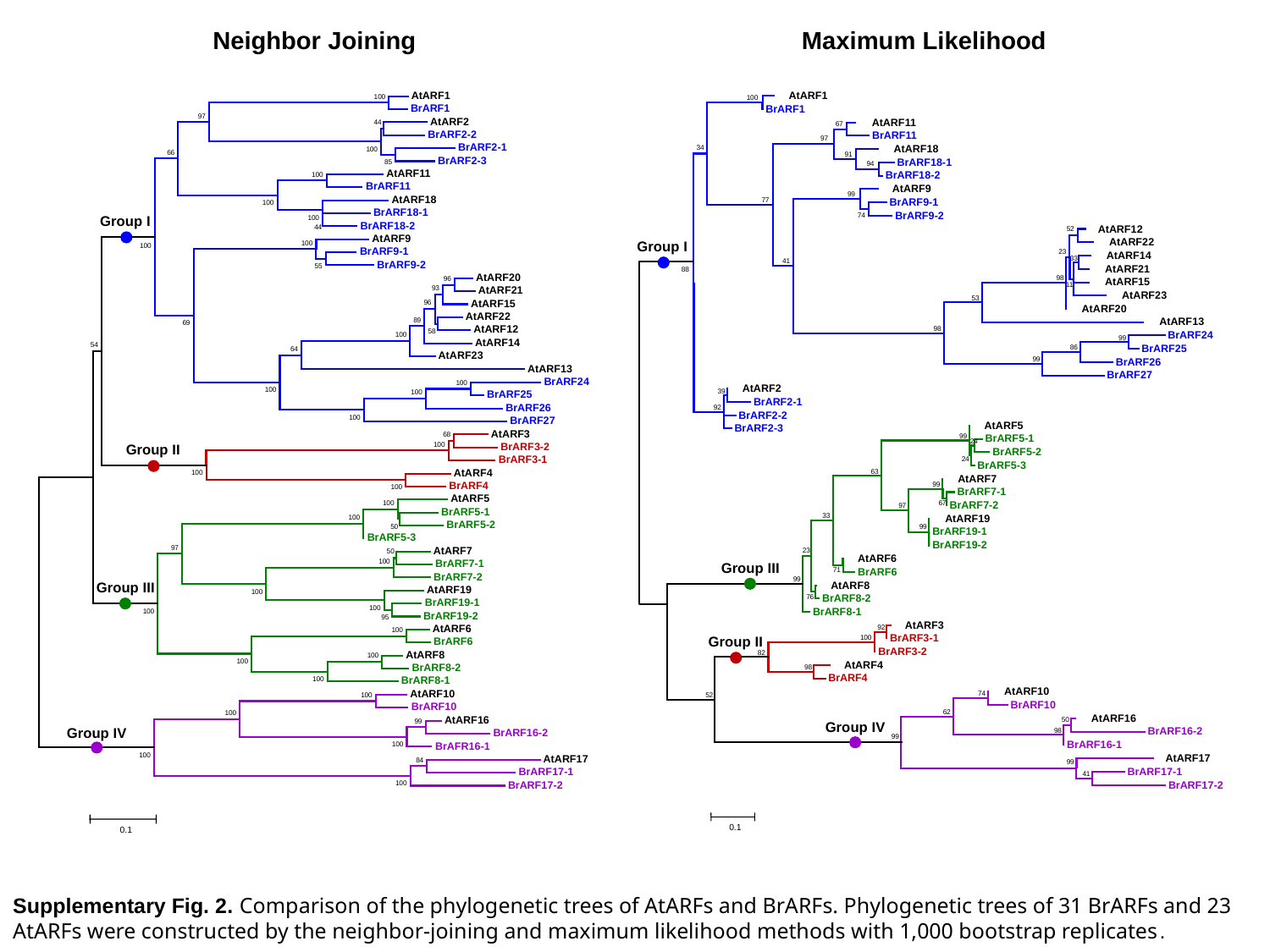

Neighbor Joining
 AtARF1
100
 BrARF1
97
 AtARF2
44
 BrARF2-2
 BrARF2-1
100
66
 BrARF2-3
85
 AtARF11
100
 BrARF11
 AtARF18
100
 BrARF18-1
100
 BrARF18-2
44
 AtARF9
100
100
 BrARF9-1
 BrARF9-2
55
 AtARF20
96
 AtARF21
93
 AtARF15
96
 AtARF22
89
69
 AtARF12
58
100
 AtARF14
54
64
 AtARF23
 AtARF13
 BrARF24
100
100
 BrARF25
100
 BrARF26
100
 BrARF27
 AtARF3
68
100
 BrARF3-2
 BrARF3-1
 AtARF4
100
 BrARF4
100
 AtARF5
100
 BrARF5-1
100
 BrARF5-2
50
 BrARF5-3
97
 AtARF7
50
100
 BrARF7-1
 BrARF7-2
 AtARF19
100
 BrARF19-1
100
100
 BrARF19-2
95
 AtARF6
100
 BrARF6
 AtARF8
100
100
 BrARF8-2
 BrARF8-1
100
 AtARF10
100
 BrARF10
100
 AtARF16
99
 BrARF16-2
 BrAFR16-1
100
100
 AtARF17
84
 BrARF17-1
 BrARF17-2
100
Group I
Group II
Group III
Group IV
0.1
Maximum Likelihood
 AtARF1
100
BrARF1
 AtARF11
67
BrARF11
97
 AtARF18
34
91
BrARF18-1
94
BrARF18-2
 AtARF9
99
BrARF9-1
77
BrARF9-2
74
AtARF12
52
Group I
 AtARF22
23
 AtARF14
33
41
 AtARF21
88
98
 AtARF15
11
 AtARF23
53
 AtARF20
 AtARF13
98
BrARF24
99
BrARF25
86
99
BrARF26
BrARF27
 AtARF2
39
BrARF2-1
92
BrARF2-2
 AtARF5
BrARF2-3
99
BrARF5-1
24
BrARF5-2
24
BrARF5-3
63
 AtARF7
99
BrARF7-1
67
BrARF7-2
97
33
 AtARF19
99
BrARF19-1
BrARF19-2
23
 AtARF6
Group III
BrARF6
71
99
 AtARF8
BrARF8-2
76
BrARF8-1
 AtARF3
92
Group II
BrARF3-1
100
BrARF3-2
82
 AtARF4
98
BrARF4
 AtARF10
74
52
BrARF10
62
 AtARF16
Group IV
50
BrARF16-2
98
99
BrARF16-1
AtARF17
99
BrARF17-1
41
BrARF17-2
0.1
Supplementary Fig. 2. Comparison of the phylogenetic trees of AtARFs and BrARFs. Phylogenetic trees of 31 BrARFs and 23 AtARFs were constructed by the neighbor-joining and maximum likelihood methods with 1,000 bootstrap replicates.
