# Supplementary material for: Auxin response factor gene family in Brassica rapa: genomic organization, divergence, expression, and evolution
Source: Mol Genet Genomics. 2012 Aug 24;287(10):765–84. doi: 10.1007/s00438-012-0718-4 (PMC3459075; doi:10.1007/s00438-012-0718-4)
Supplement: Supplementary file 2 — Supplementary material 2 (DOCX 24 kb) [file 438_2012_718_MOESM2_ESM.docx]

**Supplementary Table 1** Ks and Ka estimates between the *BrARF24*, *25*, *26*, and *27* genes

| Ka  Ks | *BrARF24* | *BrARF25* | *BrARF26* | *BrARF27* |
| --- | --- | --- | --- | --- |
| *BrARF24* |  | 0.06 | 0.12 | 0.24 |
| *BrARF25* | 0.11 |  | 0.13 | 0.20 |
| *BrARF26* | 0.28 | 0.29 |  | 0.21 |
| *BrARF27* | 0.45 | 0.43 | 0.42 |  |

**Supplementary Table 2** Ks and Ka estimates between the *AtARF12*, *13*, *14*, *15*, *20*, *21*, *22*, and *23* genes

| Ks  Ka | *AtARF12* | *AtARF13* | *AtARF14* | *AtARF15* | *AtARF20* | *AtARF21* | *AtARF22* | *AtARF23* |
| --- | --- | --- | --- | --- | --- | --- | --- | --- |
| *AtARF12* |  | 0.68 | 0.11 | 0.10 | 0.10 | 0.09 | 0.11 | 0.15 |
| *AtARF13* | 0.30 |  | 0.67 | 0.70 | 0.70 | 0.69 | 0.67 | 0.60 |
| *AtARF14* | 0.07 | 0.31 |  | 0.09 | 0.09 | 0.08 | 0.11 | 0.14 |
| *AtARF15* | 0.05 | 0.30 | 0.06 |  | 0.07 | 0.05 | 0.09 | 0.16 |
| *AtARF20* | 0.05 | 0.30 | 0.06 | 0.04 |  | 0.05 | 0.10 | 0.14 |
| *AtARF21* | 0.05 | 0.31 | 0.06 | 0.04 | 0.02 |  | 0.08 | 0.13 |
| *AtARF22* | 0.04 | 0.30 | 0.06 | 0.04 | 0.05 | 0.05 |  | 0.15 |
| *AtARF23* | 0.05 | 0.30 | 0.07 | 0.06 | 0.07 | 0.07 | 0.07 |  |

**Supplementary Table 3** Ks and Ka estimates for the paralog pairs of *BrARF* genes

| Group | Paralog pairs | Ks | Ka |
| --- | --- | --- | --- |
| I | *BrARF2-1* : *BrARF2-2* | 0.42 | 0.08 |
|  | *BrARF2-1* : *BrARF2-3* | 0.33 | 0.08 |
|  | *BrARF2-2* : *BrARF2-3* | 0.43 | 0.09 |
|  | *BrARF9-1* : *BrARF9-2* | 0.35 | 0.06 |
|  | *BrARF18-1* : *BrARF18-2* | 0.28 | 0.08 |
| II | *BrARF3-1* : *BrARF3-2* | 0.40 | 0.07 |
| III | *BrARF5-1* : *BrARF5-2* | 0.41 | 0.07 |
|  | *BrARF5-1* : *BrARF5-3* | 0.33 | 0.02 |
|  | *BrARF5-2* : *BrARF5-3* | 0.33 | 0.02 |
|  | *BrARF7-1* : *BrARF7-2* | 0.33 | 0.05 |
|  | *BrARF8-1* : *BrARF8-2* | 0.70 | 0.13 |
|  | *BrARF19-1* : *BrARF19-2* | 0.37 | 0.04 |
| IV | *BrARF16-1* : *BrARF16-2* | 0.43 | 0.10 |
|  | *BrARF17-1* : *BrARF17-2* | 0.68 | 0.16 |

**Supplementary Table 4** Putative auxin response elements in the promoter of the *BrARF* genes. 1 kb promoter region from 5’-UTR of the *BrARF* genes was searched by PLACE (www.dna.affrc.go.jp/PLACE) and the genomic positions of elements from the start codon of each gene were presented

| Gene | Position of putative auxin response element | | | | |
| --- | --- | --- | --- | --- | --- |
|  | S000024^a^ | S000270^b^ | S000273^c^ | S000370^d^ | S000391^e^ |
| *BrARF1* | -1186 |  | -393 | -468 |  |
| *BrARF2-1* | -1828 |  |  |  |  |
| *BrARF2-2* |  |  |  | -1225 |  |
| *BrARF2-3* | -1152 |  | -1272 |  | -854, -638 |
| *BrARF3-1* | -807 |  | -1553 | -827 |  |
| *BrARF3-2* | -1494, -719 |  | -1252 |  |  |
| *BrARF4* |  | -1564, -958 | -1605, -1504 |  |  |
| *BrARF5-1* |  | -1967, -1872 |  |  |  |
| *BrARF5-2* | -1196 | -1677 | -1122, -931 |  |  |
| *BrARF5-3* |  | -1965 |  |  |  |
| *BrARF6* | -723, -591, -478 |  | -1024 |  | -464 |
| *BrARF7-1* | -1039 | -1632 | -1647, -363, -357 | -940 |  |
| *BrARF7-2* | -308, -187 |  | -623 | -406, -94 |  |
| *BrARF8-1* |  | -1468, -1389 |  |  |  |
| *BrARF8-2* | -1627, -1346 |  |  |  |  |
| *BrARF9-1* | -682 | -1100 | -1147, -893 |  |  |
| *BrARF9-2* | -377, -360 |  | -1227, -1087, -834 |  |  |
| *BrARF10* | -1928 | -1764 | -1279 |  |  |
| *BrARF11* |  | -1869 |  |  |  |
| *BrARF16-1* | -714 |  | -1889 |  |  |
| *BrARF16-2* | -1918, -1019 | -1410 |  |  |  |
| *BrARF17-1* |  |  | -787 |  |  |
| *BrARF17-2* |  |  | -851, -653, -631, -507 |  |  |
| *BrARF18-1* |  | -1682 |  |  |  |
| *BrARF18-2* | -1135, -575 |  |  | -749 |  |
| *BrARF19-1* |  |  |  |  |  |
| *BrARF19-2* | -1620 |  | -1343 |  |  |
| *BrARF24* | -278, -211 | -362, -221 |  | -660 |  |
| *BrARF25* |  |  | -836 |  |  |
| *BrARF26* | -342 | -1051, -811 |  | -402 | -805 |
| *BrARF27* | -1380 |  |  |  |  |
| ^a^S000024, transcriptional activation element by auxin | | | | | |
| ^b^S000270, ARF binding site | | | | | |
| ^c^S000273, auxin induction element | | | | | |
| ^d^S000370, auxin responsive element found in Small Auxin-Up RNA gene promoter | | | | | |
| ^e^S000391, auxin response element | | | | | |
